# Human lung and monocyte-derived macrophages differ with regard to the effects of β_2_-adrenoceptor agonists on cytokine release

**DOI:** 10.1186/s12931-017-0613-y

**Published:** 2017-06-21

**Authors:** Tatiana Victoni, Hélène Salvator, Charlotte Abrial, Marion Brollo, Luis Cristovão Sobrino Porto, Vincent Lagente, Emmanuel Naline, Stanislas Grassin-Delyle, Philippe Devillier

**Affiliations:** 1 Laboratory of Histocompatibility and Cryopresevation, Laboratory of Tissue Repair, Rio de Janeiro, Brazil; 2 Laboratory of Research in Respiratory Pharmacology–UPRES EA220, UFR Sciences de la Santé Simone Veil, Université Versailles Saint-Quentin, Université Paris-Saclay, 11, rue Guillaume Lenoir, F-92150 Suresnes, France; 3 Department of Airway Diseases, Foch Hospital, Suresnes, France; 4 INSERM UMR1173 & Mass Spectrometry Facility, UFR Sciences de la Santé Simone Veil, Université Versailles Saint-Quentin, Université Paris-Saclay, Montigny-le-Bretonneux, France; 5 Nutrition Metabolisms and Cancer, INSERM, INRA, Université Rennes 1, Université Bretagne Loire, Rennes, France

**Keywords:** β_2_-adrenoceptor, Cytokines, Lipopolysaccharide, Lung macrophage, Monocyte-derived macrophage

## Abstract

**Background:**

β_2_-adrenoceptor agonists have been shown to reduce the lipopolysaccharide (LPS)-induced cytokine release by human monocyte-derived macrophages (MDMs). We compare the expression of β_2_-adrenoceptors and the inhibitory effect of formoterol and salmeterol on the LPS-induced release of tumor necrosis factor (TNF)-*α*, interleukin (IL)-1β, IL-6 and a range of chemokines (CCL2, 3, 4, and IL-8) by human lung macrophages (LMs) and MDMs.

**Methods:**

LMs were isolated from patients undergoing resection and MDMs were obtained from blood monocytes in the presence of GM-CSF. LMs and MDMs were incubated in the absence or presence of formoterol or salmeterol prior to stimulation with LPS. The effects of formoterol were also assessed in the presence of the phosphodiesterase inhibitor roflumilast.

**Results:**

LPS-induced cytokine production was higher in LMs than in MDMs. Salmeterol and formoterol exerted an inhibitory effect on the LPS-induced production of TNF-*α*, IL-6, CCL2, CCL3, and CCL4 in MDMs. In contrast, the β_2_-adrenoceptor agonists were devoid of any effect on LMs - even in the presence of roflumilast. The expression of β_2_-adrenergic receptors was detected on Western blots in MDMs but not in LMs.

**Conclusions:**

Concentrations of β_2_-adrenoceptor agonists that cause relaxation of the human bronchus can inhibit cytokine production by LPS-stimulated MDMs but not by LMs.

**Electronic supplementary material:**

The online version of this article (doi:10.1186/s12931-017-0613-y) contains supplementary material, which is available to authorized users.

## Background

Pollens, house dust mites (HDMs), and cat dander are major triggers in allergic respiratory diseases such as asthma [1–3]. Air pollution is also associated with the acute worsening of pre-existing asthma and chronic obstructive pulmonary disease (COPD) and with progression from asthma to COPD [4, 5].

In addition to its well-characterized involvement in the response to lipopolysaccharide (LPS), toll-like receptor 4 (TLR4) is involved in the airways’ response to various allergens (e.g. ragweed pollen, house dust extract, and cat dander) and many air pollutants including particulate matter and their components other than allergens and LPS, such as viruses and fungal spores [6–9]. Particles that are less than 5 μm in size may gain access to the lower airways and alveoli, where they encounter macrophages (which account for more than 80% of the leukocyte population) [9]. LPS-mediated activation of macrophages causes the release of cytokines (tumor necrosis factor-*α* (TNF-*α*), interleukin (IL)-1β and chemokines such as CCL2, CCL3, CCL4, and CXCL8 (IL-8)). This release contributes to airway inflammation by increasing the recruitment of inflammatory cells [10]. Recent research has highlighted the role of neutrophil recruitment in the response to allergen exposure and the subsequent development of allergen sensitization and inflammation [11].

In murine models of LPS-induced lung inflammation, formoterol and salmeterol reduce the recruitment of neutrophils to the lung and inhibit the release of pro-inflammatory mediators [12, 13]. We recently described the anti-inflammatory effect of the long-acting β_2_-adrenoceptor agonist (LABA) olodaterol on (i) murine and guinea pig models of cigarette smoke- and LPS-induced lung inflammation, and (ii) LPS-induced cytokine release from explants of human lung parenchyma [14]. In a clinical setting, salmeterol also reduces neutrophil influx, neutrophil degranulation and TNF-α release after LPS inhalation by healthy individuals [15].

β_2_-adrenoceptors are widely expressed throughout the lung [16, 17], and are found on epithelial and bronchial smooth muscle cells, endothelial and vascular smooth muscle cells, and pneumocytes [18, 19]. The β_2_-adrenoceptors expressed by airway smooth muscle are involved in the relaxant effect of β_2_-adrenoceptor agonists. Moreover, β_2_-adrenoceptors are expressed on inflammatory cells, such as neutrophils, monocytes/macrophages and lymphocytes [20–23]. With respect to the monocyte/macrophage lineage, β_2_-adrenoceptor activation was shown to variably reduce the LPS-stimulated release of leukotriene B4 (LTB4), TNF-α, IL-1β, IL-8 and CCL3 from human peripheral blood mononuclear cells [24–29]. Formoterol and salmeterol suppressed the LPS-induced release of TNF-*α* by monocyte-derived macrophages (MDMs) [30]. Clenbuterol and terbutaline suppressed the LPS-induced TNF-α and IL-6 release by phorbol-myristate-acetate-differentiated U937 human macrophages [31]. Furthermore, salmeterol reduced the cigarette-smoke-extract-induced release of IL-8 by MDMs [32].

Human monocytes, MDMs and U937 macrophages are all surrogate cell models that do not adequately recapitulate the biology of primary tissue macrophages. Previous studies have identified a large number of differentially expressed proteins [33, 34] and genes [35, 36] when comparing unstimulated monocytes, MDMs and human lung macrophages (LMs). It is noteworthy that the scarce data on the anti-inflammatory effects of β_2_-adrenoceptor agonists are much less clear for LMs than for MDMs or monocytes. The non-selective β_2_-adrenergic agonist isoprenaline did not alter the zymosan- and IgE-triggered release of the eicosanoids LTB_4_ and thromboxane B_2_ (TXB_2_) [37], whereas high concentrations of salmeterol inhibited the release of TXB_2_ in LMs [38]. Neither the short-acting β_2_-adrenergic agonists salbutamol and terbutaline nor the LABAs salmeterol and formoterol inhibit the LPS-stimulated release of IL-1β [39]. However, treatment with isoprenaline was associated with an increase in levels of cyclic AMP (cAMP) in LMs via the activation of β_2_-adrenergic receptors [22, 40, 41]. Furthermore, other cAMP-elevating agents (adenosine receptor agonists, phosphodiesterase 4 (PDE4) inhibitors, PGE_1/2/4_ and forskolin) either increased the cAMP content [22, 40] or had inhibitory effects on LPS-induced cytokine release by LMs [41–44]. During the preparation of the present manuscript, Gill et al. reported on the inhibitory effects of high concentrations of β_2_-adrenoceptor agonists on the LPS-induced production of TNF-α and IL-6 by LMs [45].

Hence, the present study was designed to assess and compare the effects of the LABAs formoterol and salmeterol on LPS-stimulated cytokine production and the expression of β_2_-adrenoceptors by LMs and MDMs. We selected a LABA concentration range (10^−11^ to 10^−7^ M) that relaxes isolated human bronchus [46, 47], and we used an LPS preparation that is selective for TLR4. We assessed the production of TNF-α, IL-6 and three CC chemokines (CCL2, CCL3, and CCL4), levels of which are markedly increased by LPS exposure and inhibited by cAMP-elevating agents [30, 41–45]. Furthermore, we assessed the LABAs’ effects on the LPS-induced production of IL-1β and IL-8, which is only weakly or not altered by various cAMP-elevating agents [30, 39, 42–44].

## Methods

### Reagents

Penicillin-streptomycin, dimethyl sulfoxide (DMSO), fetal calf serum (FCS), LPS from *Escherichia coli* (serotype 0111:B4), trypan blue dye, indomethacin, PGE_2_, salmeterol xinafoate, and formoterol fumarate were purchased from Sigma (St. Louis, MO, USA). Acrylamide, SDS, Tris, HEPES, RPMI 1640 medium, phosphate-buffered saline (PBS) and bovine serum albumin (BSA) were obtained from Eurobio Biotechnology (Les Ulis, France). Roflumilast was synthesized by Nycomed GmbH (Konstanz, Germany; a gift from Dr. H. Tenor). Recombinant human GM-CSF (rhGM-CSF) was purchased from R&D Systems Europe (Lille, France). All cell culture plastics were from CML (Nemours, France). Specific antibodies against β_2_-adrenoceptors and β-actin were obtained from Thermo Scientific (Vilnius, Lithuania) and Cytoskeleton (Denver, CO, USA), respectively. A Bradford protein assay and Precision Plus Protein Dual Color Standards were purchased from Bio-Rad (Hercules, CA, USA). Stock solutions of roflumilast and indomethacin were prepared in DMSO. A PGE_2_ stock solution (10 mM) was prepared in ethanol. All subsequent dilutions were prepared daily in complete medium. The DMSO concentration applied to cells in culture never exceeded 0.1%. Neither the vehicle nor any of the compounds used in this study altered cell viability. All wells were run in duplicate for each series of experiments performed with LMs or MDMs obtained from a single patient’s sample.

### Isolation and culture of human LMs and MDMs

Experiments on human tissues had been approved by the regional independent ethics committee (*Comité de Protection des Personnes Ile de France VIII*, Boulogne-Billancourt, France).

Lung tissue was obtained from 15 patients (mean ± standard error mean (SEM) age: 67 ± 4 years; gender (M:F): 10:5; FEV1/FVC ratio: 0.83 ± 0.04; 9 smokers and 6 ex-smokers; pack years: 47 ± 7) undergoing surgical resection for lung carcinoma and who had not received chemotherapy or radiotherapy. Only one donor was treated on a daily basis with a β_2_-adrenergic agonist. The LMs were isolated from lung parenchyma, as previously described [44]. The mean ± SEM adherent macrophage count was 191 ± 13 × 10^3^ per well in 24-well plates. More than 95% of the adherent cells were macrophages, as determined by May–Grünwald–Giemsa staining and CD68 immunocytochemistry. Cell viability exceeded 90%, as assessed by trypan blue dye exclusion.

The monocytes were isolated from blood, as previously described [48]. Briefly, peripheral blood mononuclear cells from nine healthy blood donors were harvested from human buffy-coat (*Etablissement Français du Sang*, Ivry-sur-Seine, France) by differential centrifugation on UNI-SEP® U-10 (Novamed, Jerusalem, Israel). The experiments were performed in compliance with the French legislation on blood donation and blood product use. Cells were resuspended in RPMI 1640 medium supplemented with penicillin 100 IU.ml^−1^-streptomycin 100 μg.ml^−1^, L-glutamine 2 mM and 10% (v/v) FCS and seeded into 24-well plates at a density of 10^6^ cells/well. Monocytes were isolated by adherence on cell culture plates for 1.5 h. Non-adherent cells were removed by aspiration, and the remaining monocytes were incubated with 50 ng.ml^−1^ rhGM-CSF for 8 days to obtain MDMs [48].

### Treatment of LMs and MDMs with salmeterol and formoterol

The experiments were performed in RPMI medium supplemented with 1% FCS. The 24-well plates containing either LMs or MDMs were washed and pre-incubated with vehicle, salmeterol or formoterol for 1 h before stimulation with LPS. Following a 24 h incubation period, supernatants were collected and stored at −80 °C for later analysis of the cytokine concentration. The submaximal LPS concentration (10 ng.ml^−1^) was selected on the basis of previous data [43, 44] [see Additional file 1].

To explore the LMs’ responsiveness to a β_2_-adrenoceptor agonist, the effect of formoterol (10 nM) was also tested in the presence (1 or 100 nM) or absence of roflumilast. Roflumilast acts as a selective PDE4 inhibitor up to a concentration of 1 μM [49]. This compound has been shown to enhance the inhibitory effect of cAMP-inducing agents (such as PGE_2_) on the LPS-induced release of cytokine by LMs [44]. In this series of experiments, PGE_2_ (10 nM) was used as an internal control for the LMs’ responsiveness to a cAMP-elevating agent [42, 44]. In order to avoid any interference of the LPS-induced production of endogenous prostanoids on the response to formoterol and PGE_2_, the experiments were performed in presence of indomethacin (1 μM) [44].

### Measurement of cytokine production

The levels of cytokine in the supernatants were measured using the Duoset® ELISA kit (R&D Systems Europe). The optical density was determined at 450 nm (MRX II, Dynex Technologies, Saint-Cloud, France). Cytokine levels were expressed in ng per 10^6^ cells. The detection limits of these assays were 4 pg.ml^−1^ for CCL3 and IL-1β, 8 pg.ml^−1^ for TNF-α, CCL2 and CCL4, 9 pg.ml^−1^ for IL-6, and 32 pg.ml^−1^ for IL-8.

### Expression of β_2_-adrenoceptors on LMs and MDMs

For real-time quantitative-PCR (RT-qPCR) analysis, LMs or MDMs (stimulated or not with LPS for 24 h) were harvested in TRIzol® reagent (Life Technologies, Saint Aubin, France). The RNA’s intactness was determined by running an aliquot of each sample on an Experion^TM^ automated electrophoresis station (Bio-Rad, Marnes-la-Coquette, France). Next, 1 μg of total RNA was reverse-transcribed using SuperScript® III First-strand SuperMix kit (Life Technologies). Specific TaqMan® arrays based on predesigned reagents (Life Technologies) were used for the analysis of β_2_-adrenoceptor transcripts (*ADRB2*). RT-qPCR was performed using Gene Expression Master Mix (Life Technologies) with 20 ng of cDNA in a StepOnePlus thermocycler (Life Technologies). The thermal cycling conditions were as follows: initial denaturation at 95 °C for 10 min, followed by 40 cycles of 95 °C for 15 s and 60 °C for 1 min. The housekeeping genes coding for hypoxanthine phosphoribosyltransferase (*HPRT1)* and glyceraldehyde-3-phosphate dehydrogenase (GAPDH) were used for signal normalization. The relative expression of mRNAs was calculated according to the 2^(−∆Ct)^ method [50].

For Western blotting, LMs and MDMs were incubated with medium alone or LPS for 24 h. The cells were then washed with PBS and lysed for 15 min in an appropriate buffer (Cytobuster, Novagen, San Diego, CA, USA) containing protease inhibitor cocktail and phosphatase inhibitor cocktail (Roche, Mannheim, Germany) on ice. Equal amounts of cell lysate (30 μg) were separated on 10% SDS-PAGE gels and then transferred onto nitrocellulose membranes. The membranes were blocked for 1 h with 5% w/v non-fat powdered milk in Tris base containing 0.1% Tween 20. Next, the membranes were incubated with a mouse monoclonal antibody specific for human β_2_-adrenoceptors (Thermo Scientific, Vilnius, Lithuania) and diluted (1/1000) for 2 h at room temperature. After washing, the membranes were incubated for 2 h with a horseradish-peroxidase-conjugated anti-mouse antibody (Dako, Glostrup, Denmark). The membranes were then incubated with an enhanced chemiluminescence solution for 1 min and quantified with QuantityOne 4.2.1 (Bio-Rad, Marnes-La-Coquette, France).

### Statistical analysis

Data were expressed as the mean ± SEM; n represents the number of patients from whom MDM or LM preparations were obtained. Wilcoxon’s test or a one-way ANOVA for repeated measures was followed by Dunnett’s post-tests, as appropriate. The threshold for statistical significance was set to *p* ≤ 0.05.

## Results

### Effects of LPS on cytokine production by MDMs and LMs

There was no significant difference between unstimulated LMs and MDMs in terms of the production of TNF-α, IL-1β, and the chemokines (IL-8, CCL2, CCL3, and CCL4). However, IL-6 production was higher in MDMs. Following incubation with LPS, the release of all cytokines other than TNF-α and CCL4 was greater for LMs than for MDMs (Table 1).

**Table 1:** The effect of LPS on cytokine levels in the supernatants of MDM and LM cultures

	Monocyte-derived macrophages		Lung macrophages	
Cytokine	LPS -	LPS +	LPS -	LPS +
TNF-α	1.8 ± 0.5	16.4 ± 6.5	0.7 ± 0.1	33.8 ± 7.1
IL-1ß	0.05 ± 0.01	0.09 ± 0.02	0.07 ± 0.03	0.54 ± 0.18*
IL-6	1.3 ± 0.5	13.2 ± 3.6	0.2 ± 0.1*	229.2 ± 80.3*
CCL2	2.2 ± 0.6	9.7 ± 2.4	3.1 ± 0.9	19.6 ± 3.9*
CCL3	4.5 ± 2.5	22.2 ± 8.1	3.1 ± 0.8	148.7 ± 41.2*
CCL4	6.5 ± 2.3	86.5 ± 31.4	6.8 ± 2.3	264.2 ± 78.6
IL-8	5.5 ± 2.3	93.2 ± 32.1	39.3 ± 14.3	1411.4 ± 383.1*

Macrophages were incubated with medium only (LPS-) or 10 ng.ml^−1^ LPS in medium (LPS+) for 24 h. Cell culture supernatants were collected and analyzed using an ELISA. Data are expressed as the mean ± SEM ng.10^−6^ cells from 5 to 8 experiments. Asterisks indicate significant differences (Wilcoxon’s t test) between MDM and LM experiments

### Effects of formoterol and salmeterol on LPS-induced cytokine release by MDMs and LMs

**Fig. 1:**
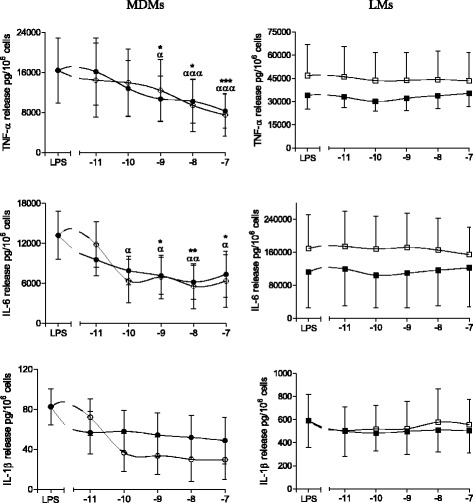
Effects of formoterol and salmeterol on LPS-induced release of TNF-α, IL-6 and IL-1β by monocyte-derived macrophages (MDMs) and lung macrophages (LMs). MDMs (*left-hand column*) and LMs (*right-hand column*) were incubated with formoterol (○,□) and salmeterol (●,■) for 1 h prior to stimulation with LPS (10 ng.ml^−1^) for 24 h. The culture supernatants were collected, and the cytokine concentrations were measured using an ELISA. The data represent the mean ± SEM of 5 to 8 independent experiments..*p < 0.05, **p < 0.01, ***p < 0.001 for salmeterol vs. LPS, ^α^ *p* < 0.05, ^αα^ *p* < 0.01, ^ααα^ *p* < 0.001 for formoterol vs. LPS

**Fig. 2:**
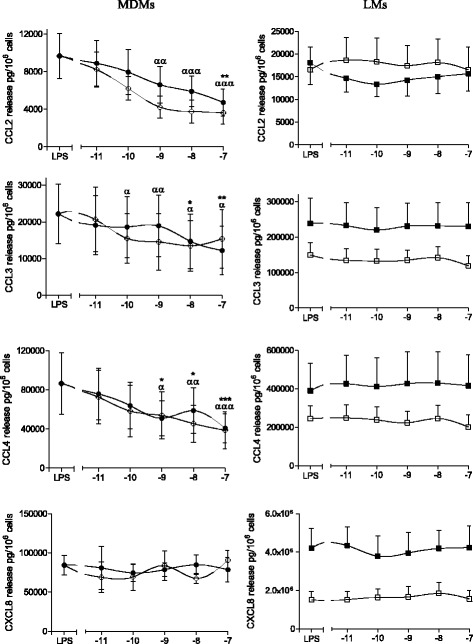
Effects of formoterol and salmeterol on LPS-induced release of chemokines by monocyte-derived macrophages (MDMs) and lung macrophages (LMs). MDMs (left-hand column) and LMs (right-hand column) were incubated with formoterol (○,□) and salmeterol (●,■) for 1 h prior to stimulation with LPS (10 ng.ml^−1^) for 24 h. The culture supernatants were collected, and the cytokine concentrations were measured using an ELISA. The data represent the mean ± SEM of 5 to 8 independent experiments. *p < 0.05, **p < 0.01, ***p < 0.001 for salmeterol vs. LPS, ^α^ *p* < 0.05, ^αα^ *p* < 0.01, ^ααα^ *p* < 0.001 for formoterol vs. LPS

We next investigated the effects of serial increases in the concentration (10^−11^ to 10^−7^ M) of formoterol and salmeterol on LPS-induced cytokine release. In MDMs, formoterol and salmeterol inhibited the LPS-induced production of TNF-α, IL-6 and the three CCL chemokines at concentrations greater than or equal to 10^−10^ M (Figs. 1 and 2). The respective effects of formoterol and salmeterol on the (weak) production of IL-1β were highly variable from one preparation to another. The production of IL-8 was not altered by the two LABAs. In sharp contrast to the results for MDMs, formoterol and salmeterol did not alter the LPS-induced production of any of the seven cytokines by LMs (Figs. 1 and 2). To definitively establish that the LMs’ lack of response is not restricted to these two LABAs, we performed additional experiments on four preparations of MDMs and LMs with salbutamol at 1 μM (a concentration that causes maximal relaxation of isolated human bronchus and is equipotent to the concentrations used in the present study with the LABAs (0.01 μM for formoterol and 0.1 μM for salmeterol)). Our results confirmed that this short-acting β_2_-adrenoceptor agonist inhibited MDMs (to much the same extent as in the work by Gill et al. [45]) but had no effect on LMs [see Additional file 2].

### Effect of formoterol on LPS-induced cytokine release in the presence of roflumilast

**Fig. 3:**
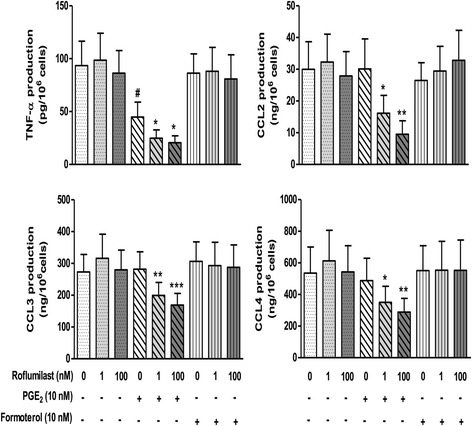
Effects of formoterol, PGE_2_ and roflumilast on LPS-induced TNF-α, CCL2, CCL3 and CCL4 release from LMs. Cells were pre-incubated with indomethacin (1 μM) for 30 min, followed by incubation with roflumilast (1 nM or 100 nM), PGE_2_ (10 nM), formoterol (10 nM) or vehicle for another 30 min prior to stimulation with LPS (10 ng.ml^−1^) for 24 h). The data represent the mean ± SEM of 6 different experiments, *p < 0.05, **p < 0.01, ***p < 0.001 vs. LPS + PGE2 treatment; #*p* < 0.05 vs. LPS

Since formoterol was more potent than salmeterol in altering LPS-induced cytokine production by MDMs, we also looked at whether the presence of roflumilast could unmask an effect of 10^−8^ M formoterol on the LPS-induced release of TNF-α and the CCL chemokines by LMs. In this series of experiments, formoterol has no effect alone or in the presence of roflumilast on LPS-induced release of TNF-α, CCL2, CCL3 and CCL4 (Fig. 3). In contrast, the greater inhibitory effect of PGE_2_ on production of the four cytokines in the presence of roflumilast evidences the latter drug’s effect on a cAMP-elevator other than the LABAs in LMs (Fig. 3). In addition, formoterol did not increase significantly the inhibitory effect of PGE_2_ (data not shown).

### Expression of β_2_-adrenoceptors on MDMs and LMs in presence and absence of LPS

Levels of ß_2_-adrenoceptor transcript expression were similar in MDMs and LMs (Table 2), whereas β_1_- and β_3_-adrenoceptor transcripts were only found in macrophages from two and three patients, respectively (data not shown). Strikingly, incubation of LMs with LPS for 24 h induced an approximately 7-fold decrease in β_2_-adrenoceptor expression (Table 2).

**Table 2:** Expression of *β*_2_-adrenoreceptor mRNA transcripts (*ADRB2*) in human MDMs and LMs

	Relative expression in control (LPS-)	Relative expression after LPS exposure	Fold-change for LPS versus control
MDMs	172.6 [134.2, 228.7]	108.6 [76.2, 158.4]	−1.6
LMs	310.8 [187.1, 546.0]	44.7 [23.6, 75.3]	−7.5

The quoted result is the median [min, max] × 1000 of 3 to 5 independent experiments

**Fig. 4:**
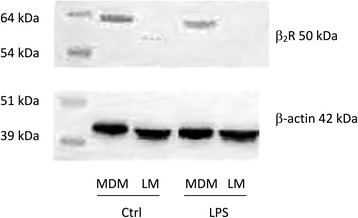
Western blot analysis of the expression of β_2_-adrenoceptors on MDMs and LMs. MDMs and LMs were incubated with medium alone (control) or LPS (10 ng.ml^−1^) for 24 h. Cell lysates were immunoblotted with a β_2_-adrenoceptor-specific antibody

To determine whether the LMs’ absence of response to the β_2_-adrenoceptor agonists was related to a loss of β_2_-adrenoceptors relative to MDMs, we performed a Western blot analysis. As shown in Fig. 4, MDMs (but not LMs) expressed β_2_-adrenoceptors. LPS treatment for 24 h did not alter the expression of the β_2_-adrenoceptors in MDMs.

## Discussion

Our present results notably showed that (i) cytokine production in response to LPS differs in MDMs and LMs, (ii) salmeterol and formoterol exert an inhibitory effect on the LPS-induced production of TNF-α, IL-6, CCL2, CCL3, and CCL4 by MDMs, (iii) the two LABAs were strikingly devoid of any effect on LMs, and (iv) Western blots revealed β_2_-adrenergic receptor in MDMs but not in LMs.

We confirmed the recent report in which formoterol and salmeterol can inhibit the LPS-induced release of TNF-α and IL-6 from MDMs [30]. We extended these findings to three CCL chemokines (CCL2, CCL3 and CCL4) involved in the recruitment of monocytes, immature dendritic cells and T cells [51–53]. The range of concentrations at which these two LABAs influence LPS-induced cytokine production is suggestive of a β_2_-adrenoceptor-dependent mechanism; this is also suggested by the attenuating effect of a β_2_-adrenoceptor antagonist on formoterol’s inhibitory action [30]. We also confirmed that the two LABAs did not alter the LPS-induced production of IL-1β and IL-8.

In a very recent study [45], salmeterol and indacaterol were found to inhibit the LPS-induced production of TNF-α and IL-6 by human LMs. However, four other β_2_-adrenoceptor agonists (formoterol, salbutamol, terbutaline and isoprenaline) were inactive, and the inhibitory effect of the two LABAs was only observed at a concentration (10^−5^ M) that is at least 100-fold higher than those used in the present study and caused maximum relaxation of isolated human bronchi [46, 47]. These differences in the inhibitory activities of the various β_2_-adrenoceptor agonists and the high concentration of the two active LABAs used in Gill et al.’s study calls into question both the clinical relevance of these results and the involvement of a β_2_-adrenoceptor-mediated effect. It should be noted that the inhibitory effect of indacaterol was only partly reversed by a selective β_2_-adrenoceptor antagonist, and the inhibitory effect of salmeterol was not reversed [45]. Moreover, the production of TXB_2_ by LMs stimulated with either zymosan or the calcium ionophore A23187 was not inhibited by salbutamol (at concentrations up to 10^−5^ M), and the inhibitory effect of salmeterol was not blocked by propranolol - further suggesting that the effects of high concentrations of LABAs are not mediated by β_2_-adrenoceptors in LMs [38], as also reported for human monocytes [27, 38]. Furthermore, four β_2_-adrenoceptor agonists (salmeterol, formoterol, salbutamol, and terbutaline) did not alter LPS- or zymosan-induced LTB_4_ release from LMs at concentrations up to 10^−5^ M [39]. Taken as a whole, these results suggest that the inhibitory effects of β_2_-adrenoceptor agonists on LMs is weak or even null, and might only be produced at very high concentrations via a β_2_-adrenoceptor-independent mechanism. Given that macrophages express membrane-associated CD14, activation of the TLR4/MD-2 complex by LPS does not therefore require dimerization of the complex induced by LPS binding protein (LBP) [54]. Nevertheless, LBP (if required) was present in the FCS added to the culture medium. Hence, LBP and CD14 do not account for the results and conclusions of the present work.

MDMs differentiated by treatment with GM-CSF have been typically considered to be phenotypically and “behaviorally” similar to LMs [30, 32, 55]. However, recent high-throughput analyses have revealed remarkable differences in gene expression between MDMs and LMs; these differences include the transcripts for G-protein-coupled receptors [35, 36]. These results call into question the use of macrophage surrogates (such as MDMs) to mimic the behavior of LMs. Although β_2_-adrenoceptor transcript levels were similar in MDMs and LMs ([35] and the present study), we found that protein levels of these receptors (as assessed by Western blotting) were much lower in LMs than in MDMs. Western blotting based on a peroxidase-conjugated secondary antibody is probably less sensitive than radioligand binding methods for detecting receptors. Human macrophages isolated from bronchoalveolar lavages (either by elutriation or adherence to culture dishes) were found to express a moderate density of β_2_-adrenoceptors in radioligand binding studies [21, 22]. In both of the latter studies [21, 22], the β_2_-adrenoceptors appear to be functionally coupled to adenylate cyclase; exposure to high concentrations of isoprenaline (≥10^−7^ M) in the presence of the PDE inhibitor isobutylmethylxanthine resulted in increased cAMP accumulation. However, the increase in cAMP was much lower in the absence of isobutylmethylxanthine, and the two studies did not determine whether the increase in cAMP levels impacted macrophage function. It is noteworthy that in a subsequent study by one of the research groups, β_2_-adrenoceptor agonists did not alter the LPS- or zymosan-induced release of LTB_4_ from LMs [39] suggesting that the signal induced by the agonists was not strong enough to inhibit the effect of either LPS or zymosan. Furthermore, in the presence of roflumilast at a concentration that enhances the inhibitory influence of PGE_2_ on LPS-induced TNF-α and chemokine production by LMs, we found that formoterol also remained inactive. This finding suggests that stimulation of the β_2_-adrenoceptors did not increase the cAMP level enough to inhibit the production of the four cytokines. In line with these results, isoprenaline alone or in combination with a PDE inhibitor had any inhibitory effect on the release of eicosanoids induced by zymosan or IgE/anti-IgE complexes by LMs. These results for LMs contrast with the additive effects of formoterol and a PDE inhibitor (roflumilast or rolipram) in human monocytes and MDMs [30, 56].

However, forskolin inhibits TXB_2_ release [37] and PGE_2_ ([41, 42, 44] and the present study), NECA and roflumilast [43, 44] inhibit the LPS-induced production of cytokines by LMs - demonstrating that other cAMP elevators than LABAs are able to curb the production of eicosanoids or cytokines from LMs. Since the adenylyl cyclase/cAMP/cAMP-dependent protein kinase A axis stimulated by PGE_2_ reduces the LPS-induced cytokine production [42], the β_2_-adrenoceptor agonists’ lack of effect in LMs is probably due to the low expression of β_2_-adrenoceptors in these cells and thus insufficient stimulation of the pathway. The use of ten-fold lower concentrations of LPS (to markedly reduce the strength of the stimulus) unmasked a modest inhibitory effect of salbutamol on the release of TNF-α by LMs [45] - suggesting that the β_2_-adrenoceptor-dependent rise in cAMP content might be only sufficient to counteract a relatively weak inflammatory stimulus. Lastly, the absence of an inhibitory effect of formoterol in LMs (evidenced in the present study) rules out the involvement of this cell type in the inhibitory effect of this LABA [57] and olodaterol [14] on LPS-induced cytokine release by human lung explants. Macrophages have been implicated in the pathophysiology of COPD and (to a lesser degree) in the inflammatory load in asthma. However, given the absence of LABAs’ effects on LMs in vitro, macrophages are unlikely to account for these compounds’ anti-inflammatory effects.

## Conclusion

Our present results showed that concentrations of β_2_-adrenoceptor agonists that cause the relaxation of isolated human bronchus can inhibit cytokine production by LPS-stimulated MDMs but not by LPS-stimulated LMs - even in the presence of a PDE inhibitor. The LMs’ lack of response could be due to low β_2_-adrenoceptor expression and thus an insufficiently strong cAMP-dependent trigger for the LPS-induced inflammatory response, since other cAMP elevators were able to inhibit the LPS-induced responses. The present results highlighted the lack of a clinically relevant, anti-inflammatory effect of β_2_-adrenoceptor agonists on LMs.

## Additional files

Additional file 1: LPS concentration-response data for MDMs and LMs, and time-course experiments in LMs (figures). (PDF 94 kb)

Additional file 2: Effect of salbutamol (1 μM) on LPS-induced cytokine production by MDMs and LMs (figures). (PDF 52 kb)

## Abbreviations

BSA: Bovine serum albumin
cAMP: Cyclic AMP
COPD: Chronic obstructive pulmonary disease
DMSO: Dimethyl sulfoxide
FCS: Fetal calf serum
GAPDH: Glyceraldehyde-3-phosphate dehydrogenase
HDMs: House dust mites
IL: Interleukin
LMs: Human lung macrophages
LPS: Lipopolysaccharide
LTB4: Leukotriene B4
MDMs: Human monocyte-derived macrophages
PBS: Phosphate-buffered saline
PDE4: Phosphodiesterase 4
rhGM-CSF: Recombinant human GM-CSF
TLR4: Toll-like receptor 4
TNF: Tumor necrosis factor
TXB2: Thromboxane B_2_
