# Supplementary material for: Human lung and monocyte-derived macrophages differ with regard to the effects of β2-adrenoceptor agonists on cytokine release
Source: Respir Res. 2017 Jun 21;18:126. doi: 10.1186/s12931-017-0613-y (PMC5480184; doi:10.1186/s12931-017-0613-y)

Additional file 1 (.pdf):

- LPS concentration-response data for MDMs (empty bars) and LMs (filled bars) (n=5).

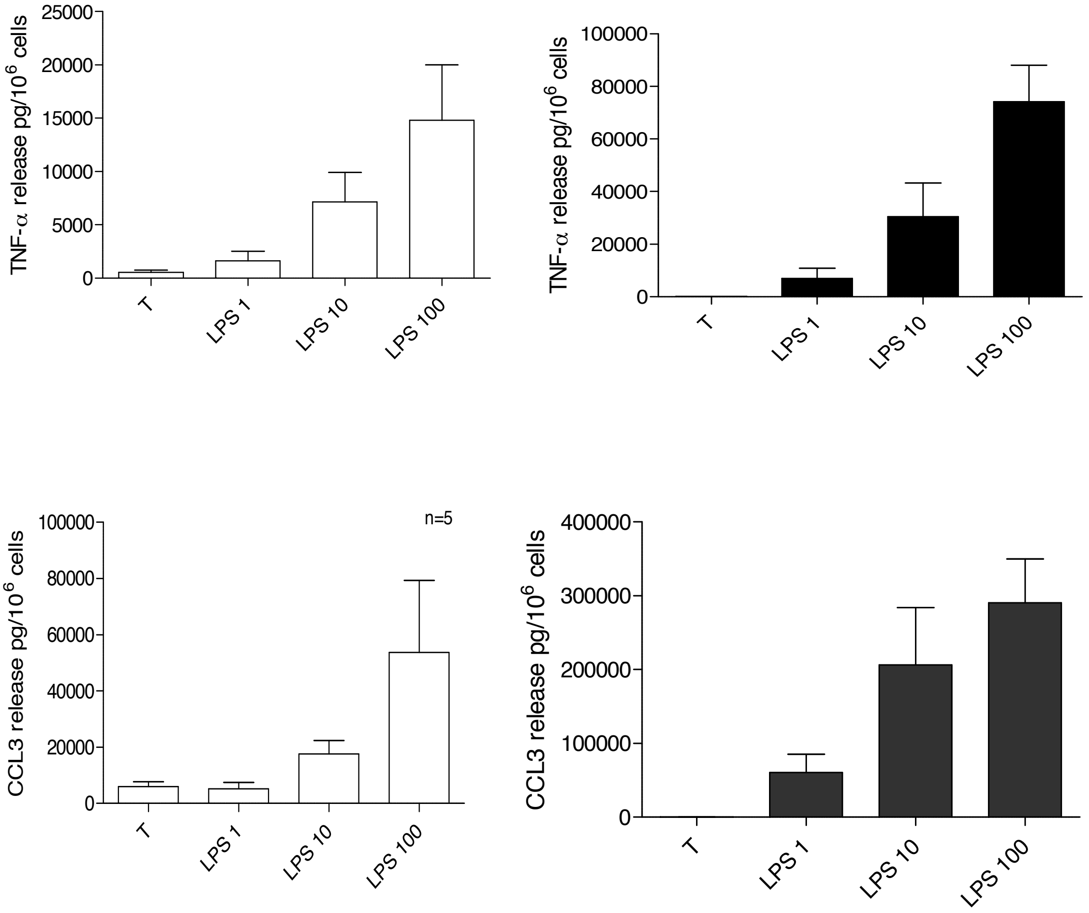

- Time-course experiments in LMs with LPS (n = 4).

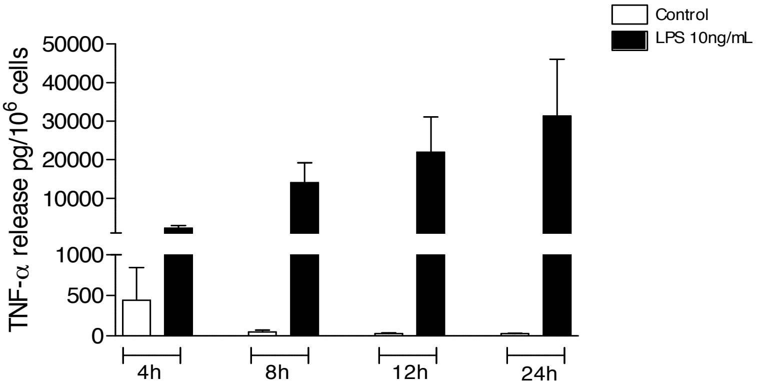

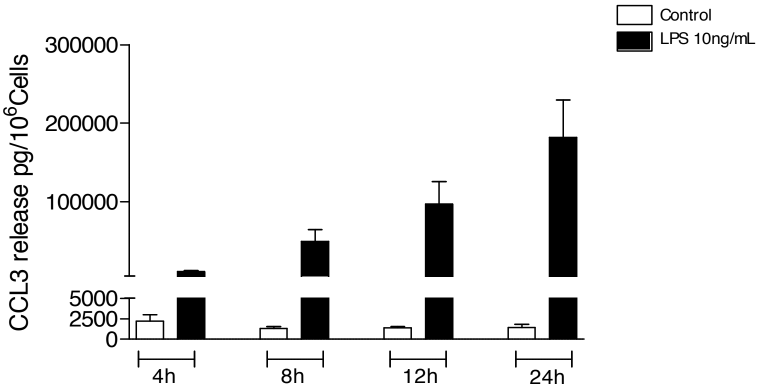

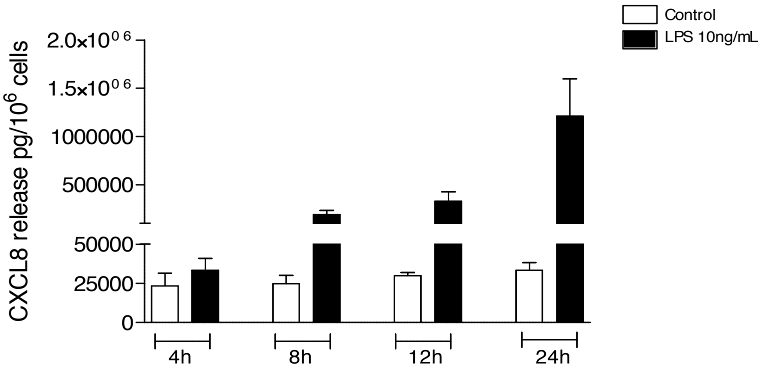

Supplement: Supplementary file 1 — LPS concentration-response data for MDMs and LMs, and time-course experiments in LMs (figures). (PDF 94 kb) [file 12931_2017_613_MOESM1_ESM.pdf]
