# Supplementary material for: Human lung and monocyte-derived macrophages differ with regard to the effects of β2-adrenoceptor agonists on cytokine release
Source: Respir Res. 2017 Jun 21;18:126. doi: 10.1186/s12931-017-0613-y (PMC5480184; doi:10.1186/s12931-017-0613-y)

Additional file 2 (.pdf):

Effect of salbutamol (1 µM) on LPS (10 ng/ml)-induced cytokine production by MDMs (empty bars) and LMs (filled bars).

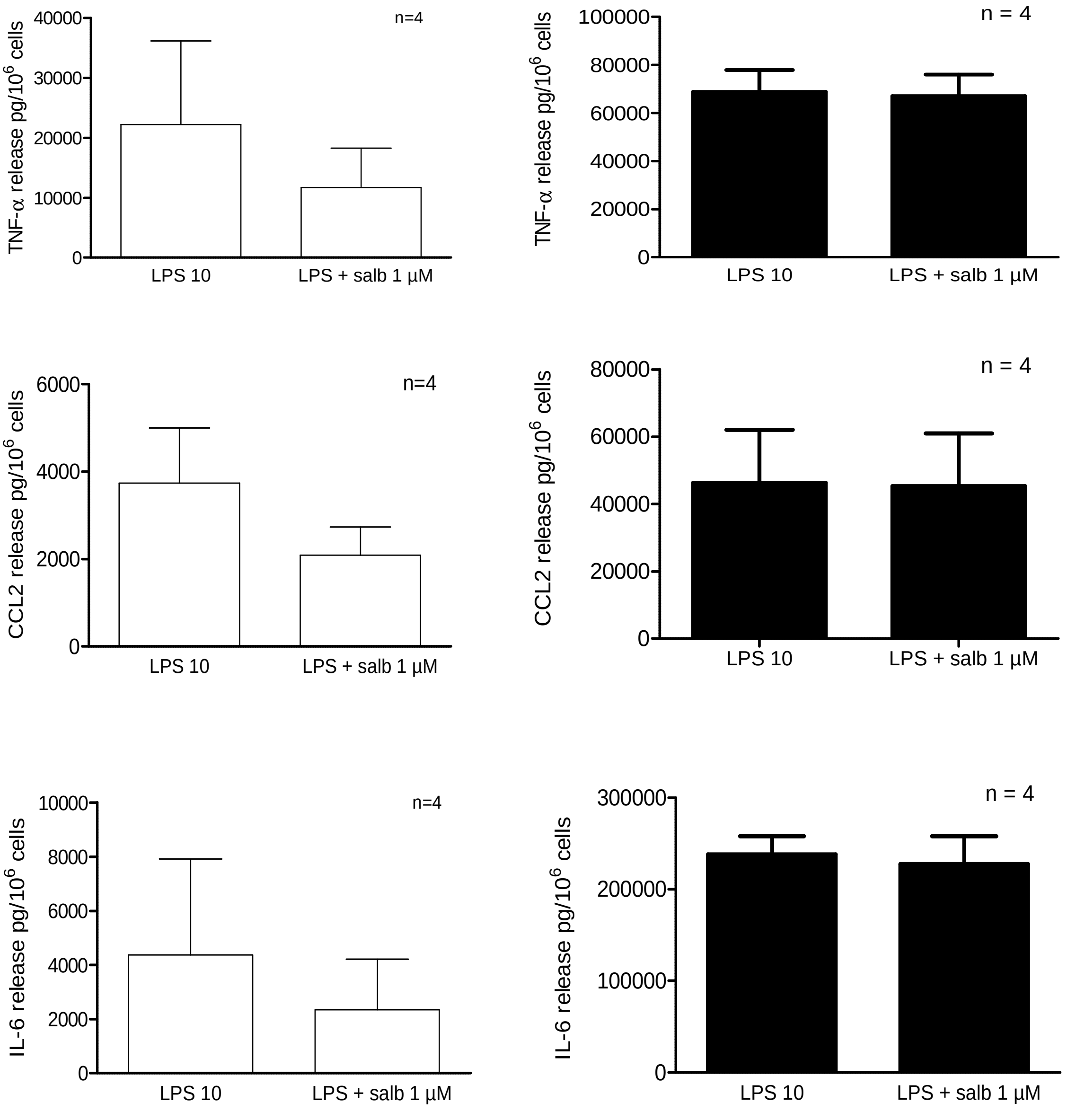

Supplement: Supplementary file 2 — Effect of salbutamol (1 μM) on LPS-induced cytokine production by MDMs and LMs (figures). (PDF 52 kb) [file 12931_2017_613_MOESM2_ESM.pdf]
